# Inability to access addiction treatment predicts injection initiation among street-involved youth in a Canadian setting

**DOI:** 10.1186/s13011-015-0046-x

**Published:** 2016-01-06

**Authors:** Kora DeBeck, Thomas Kerr, Seonaid Nolan, Huiru Dong, Julio Montaner, Evan Wood

**Affiliations:** British Columbia Centre for Excellence in HIV/AIDS, Vancouver, Canada; School of Public Policy, Simon Fraser University, Vancouver, Canada; Division of AIDS, Department of Medicine, University of British Columbia, Vancouver, Canada

**Keywords:** Injection initiation, At-risk youth, Addiction treatment, Injection prevention

## Abstract

**Background:**

Preventing injection drug use among vulnerable youth is critical for reducing serious drug-related harms. Addiction treatment is one evidence-based intervention to decrease problematic substance use; however, youth frequently report being unable to access treatment services and the impact of this on drug use trajectories remains largely unexplored. This study examines the relationship between being unable to access addiction treatment and injection initiation among street-involved youth.

**Methods:**

Data were derived from the At-Risk Youth Study (ARYS), a prospective cohort of street-involved youth aged 14–26 who use illicit drugs, from September 2005 to May 2014. An extended Cox model with time-dependent variables was used to identify factors independently associated with injection initiation.

**Results:**

Among 462 participants who were injection naïve at baseline, 97 (21 %) initiated injection drug use over study follow-up and 129 (28 %) reported trying but being unable to access addiction treatment in the previous 6 months at some point during the study period. The most frequently reported reason for being unable to access treatment was being put on a wait list. In a multivariable Cox regression analysis, being unable to access addiction treatment remained independently associated with a more rapid rate of injection initiation (Adjusted Hazard Ratio =2.02; 95 % Confidence Interval: 1.12–3.62), after adjusting for potential confounders.

**Conclusion:**

Inability to access addiction treatment was common among our sample and associated with injection initiation. Findings highlight the need for easily accessible, evidence-based addiction treatment for high-risk youth as a means to prevent injection initiation and subsequent serious drug-related harms.

## Findings

Preventing vulnerable youth from initiating injection drug use is critical for reducing drug-related morbidity and mortality [1–4]. There are a number of features of young drug injectors that highlight the urgency of intervening early in their drug use trajectories to prevent the transition to injection drug use [5]. For instance, prior research among street-involved youth indicates that once youth initiate injection drug use, the majority rapidly become established injectors [6]. Young new injection initiators are also more prone to engage in risky drug use practices that put them at higher risk of drug overdose and infectious disease transmission [3, 4, 7–9].

Structural level influences, such as homelessness and unemployment [10–12], alongside individual level factors including childhood trauma, and specific drug use patterns [13, 14], have been recognized as factors that facilitate transitions into injection drug use among vulnerable youth. While these findings suggest that injection prevention efforts should be directed to the areas of housing, employment, and childhood trauma prevention and recovery, addiction treatment may provide additional opportunities to reduce injection initiation. It has long been established that addiction treatment is one of the most cost-effective interventions to reduce problematic substance use [15, 16]. However, prior studies indicate that many vulnerable individuals are unable to access addiction treatment [17–20]. To determine the role that barriers to accessing addiction treatment may play in influencing drug use trajectories, we examined whether inability to access addiction treatment was associated with injection initiation among a cohort of street-involved youth.

## Methods

Data for this study was obtained from the At-Risk Youth Study (ARYS), which is an open prospective cohort of street-involved youth in Vancouver, Canada that has been described in detail previously [21]. In brief, study recruitment is open and undertaken using snowball sampling and extensive street-based outreach methods. To be eligible, participants at recruitment must be age 14–26 years, have used illicit drugs in the past 30 days, and provide written informed consent. At baseline and on a semi-annual basis, participants complete an interviewer-administered questionnaire that elicits information related to drug use and contact with health and social services. At each study visit participants are provided with a stipend ($30 Canadian currency) for their time. The study has been approved by the University of British Columbia’s Research Ethics Board.

The study period for this analysis was September 2005 to May 2014. To examine the potential relationship between initiation into injection drug use and inability to access addiction treatment, all participants who had never injected drugs at baseline and had completed at least one follow-up visit during the study period were included in the present analysis. The primary outcome of interest was injection initiation which was defined as the midpoint between the last report of remaining injection naïve and the first report of having used a needle to chip, fix or muscle drugs. For descriptive purposes we also assessed the median number of years between initiation of non-injection “hard” drug use (defined as use of heroin, cocaine, crack, or crystal methamphetamine) and initiation of injection drug use. These estimates were based on the reported age of first non-injection “hard” drug use, and age of participants at the midpoint between the last report of remaining injection naïve and the first report of having used a needle to chip, fix, or muscle drugs. The primary explanatory variable of interest was being unable to access addiction treatment defined as responding affirmatively to the question: "In the past 6 months, have you tried to access any treatment program but were unable?" Participants were also asked to specify the types of addiction treatment they had difficulty accessing (e.g., detox, recovery house, treatment center, counselor, other), as well as the main reason they were unable to access the program (waiting lists, behavioral issues, rejection from program, logistics such as hours of optional, location, paperwork etc.).

To determine whether there was a significant relationship between our outcome of interest and our primary explanatory variable we *a priori* selected a range of secondary explanatory variables we hypothesized might be associated with both injection initiation and being unable to access addiction treatment. Secondary explanatory factors included: number of years since initiated “hard” drug use defined as use of cocaine, crack, heroin, or crystal methamphetamine (per additional year); gender (female vs. male); ethnicity (Caucasian vs. other); non-injection cocaine use (yes vs. no); crack smoking (yes vs. no); non-injection crystal methamphetamine use (yes vs. no); and non-injection heroin use (yes vs. no). All drug use variables including being unable to access addiction treatment refer to circumstances and behaviors over the previous 6 months and were treated as time-updated covariates on the basis of semi annual follow-up data. In addition, to protect against reverse causation whereby reported behaviors were a consequence of drug injecting, all drug use variables including being unable to access addiction treatment were lagged to the previous available observation [10, 11].

To assess the relationship between being unable to access addiction treatment and injection initiation, as a first step we calculated the incidence density of injection initiation using a Poisson model. Then, using an extended Cox model with time-dependent variables, we estimated the unadjusted relative hazards and 95 % confidence intervals for factors associated with injection initiation [22]. To fit our multivariable Cox model, we ran a fixed multivariable model where all variables of interest were included into a single model. All statistical analyses were performed using SAS software version 9.3 (SAS, Cary, NC, USA). All tests of significance were two-sided.

## Results

Overall, 1157 street-involved youth were recruited into the ARYS cohort during the study period. At enrolment 659 (57 %) youth had never injected drugs. Among this group, during the study period, the average yearly loss to follow-up rate was 3.15 %. At the time the analysis was conducted, 462 (70 %) participants completed at least one study follow-up to assess for injection initiation and were therefore included in the analysis. There were no significant differences with respect to gender (Chi-square *p-*value =0.943; degrees of freedom [df] =1) or ethnicity (Chi-square *p-*value =0.117; df =1) between the 462 youth who represented the eligible study population and the 197 injecting naïve youth who were ineligible because they either did not have a follow up visit at the time the analysis was conducted or were not enrolled in the cohort long enough to be due for a study follow-up.

Among the sample of 462 youth included in the study, 142 (31 %) were female and the median age was 21.5 years (interquartile range [IQR] = 19.6–23.2). The median number of study visits was 4 (IQR = 2–6), the median time between study visits was 6.2 (IQR: 5.7–8.1) months, and the median follow up time per participant was 22.4 (IQR = 11.9–43.2) months. Baseline characteristics of the study sample are presented in Table 1. Over study follow-up, 97 (21 %) injection initiation events were observed for an incidence density of 8.6 cases per 100 person years [95 % Confidence Interval (CI): 7.0–10.6]. The median time to injection initiation from study enrolment was 11.2 months (IQR: 3.9–23.9), and the median number of years between initiation of non-injection “hard” drug use (defined as use of heroin, cocaine, crack, or crystal methamphetamine) and initiation of injection drug use was 7.1 (IQR = 4.6–9.5).

**Table 1:** Baseline characteristics and Cox regression analysis for factors associated with injection initiation among street-involved youth (*n* = 462)

Characteristic	Baseline Characteristics		Bivariable and Multivariable Cox Regression Analysis			
	Injection Initiation		Unadjusted HR^a^	*p*-value	Adjusted HR (95 % CI)	*p*-value^f^
	Yes (*n* = 97) n (%)	No (*n* = 365) n (%)	(95 % CI)^b^			
Unable to access addiction treatment ^d,e^						
Yes	15 (15.5)	33 (9.0)	2.19 (1.27–3.78)	0.005	2.02 (1.12–3.62)	0.019
No	80 (82.5)	324 (88.8)				
Years since initiated hard drug use (HR per additional year)						
Median	5.4	5.4	1.00 (0.94–1.07)	0.893	0.99 (0.92–1.06)	0.714
IQR	(3.7–7.8)	(3.1–7.9)				
Caucasian Ethnicity						
Yes	68 (70.1)	219 (60.0)	1.50 (0.97–2.31)	0.069	1.40 (0.88–2.21)	0.152
No	29 (29.9)	146 (40.0)				
Female Gender						
Yes	28 (28.9)	114 (31.2)	0.96 (0.62–1.50)	0.872	1.06 (0.68–1.65)	0.805
No	69 (71.1)	251 (68.8)				
Heroin Use^c,d,e^						
Yes	24 (24.7)	52 (14.2)	2.12 (1.34–3.36)	0.001	1.48 (0.86–2.55)	0.157
No	70 (72.2)	307 (84.1)				
Cocaine Use^c,d,e^						
Yes	43 (44.3)	186 (51.0)	1.17 (0.77–1.78)	0.449	1.06 (0.69–1.64)	0.782
No	52 (53.6)	176 (48.2)				
Crack Smoking^d,e^						
Yes	68 (70.1)	190 (52.1)	1.71 (1.11–2.63)	0.015	1.23 (0.76–1.97)	0.402
No	27 (27.8)	171 (46.8)				
Crystal Meth Use^c,d,e^						
Yes	51 (52.6)	122 (33.4)	2.31 (1.53–3.47)	<0.001	2.00 (1.32–3.04)	0.001
No	43 (44.3)	238 (65.2)				

Not all cells add up to 462 as participants may choose not to answer sensitive questions

^a^ *HR* hazard ratio; ^b^ *CI* confidence interval

^c^denotes non-injection use; ^d^denotes activities in the 6 months prior to follow-up interview; ^e^refers to the activities lagged to the pervious available study follow-up; ^f^ *p*-values based on Wald test

At some point during the study period 129 (28 %) youth reported being unable to access addiction treatment. In total, 183 study observations included a report of being unable to access addiction treatment. Among these study observations, the most common type of addiction treatment that participants reported being unable to access was detox services (*n* = 76, 41 %), followed by treatment centers (*n* = 65, 35 %), recovery houses (*n* = 20, 10 %), and counselors (*n* = 8, 4 %). The main reason participants reported being unable to access addition treatment was waiting lists (*n* = 118, 66 %), followed by logistical issues such as hours of optional, location, required paperwork etc. (*n* = 32, 18 %). Being rejected from the program for an unspecified reason (*n* = 16, 9 %), and having behavioral issues (*n* = 10, 6 %) were two other common barriers. Note, out of the 183 observations that included a report of being unable to access addiction treatment, 10 observations did not specify the type of treatment that the participant was unable to access, 162 observations indicated one type of treatment, and 11 observations indicated 2 types of treatment. Similarly, 11 observations did not specify a reason the participant was unable to access treatment, 164 observations indicated one reason, and 8 observations indicated 2 reasons.

Table 1 shows the unadjusted and adjusted relative hazards of injection initiation. Being unable to access addiction treatment was significantly associated with injection initiation in both bivariable [hazard ratio =2.19, 95 % CI: 1.27–3.78] and multivariable Cox regression analyses [adjusted hazard ratio =2.02, 95 % CI: 1.12–3.62].

## Discussion

Among our sample of youth, 28 % sought but were unable to access addiction treatment at some point during the study period. Youth who were unable to access addiction treatment were over two times more likely to subsequently initiate injection drug use, highlighting a critical missed opportunity to intervene to prevent injection initiation among high-risk youth. These findings are consistent with prior studies indicating that inability to access and engage with key health and social services, such as addiction treatment, housing, and employment negatively influences drug use behaviors and trajectories among vulnerable populations [10–12, 19, 23]. Prior cross-sectional analyses also found that contact with addiction treatment significantly delayed injection initiation among heroin users in the United States, highlighting the protective benefits of addiction treatment [24].

Given the importance of intervening early in youths’ drug use trajectories to prevent injection initiation, our findings indicate that addressing deficiencies in youth addiction treatment, particularly with respect to waiting lists and logistical issues, should be a top priority. Numerous barriers to accessing addiction treatment have been identified in the literature and include: limited availability and insufficient use of evidence-based medication-assisted therapies; long wait times; lack of adequately trained providers; age restrictions; limited hours of operation; discrimination; and stigma, among others [18, 19, 25–31].

Our study has limitations. First, as with other studies of street-involved youth, the ARYS cohort is not a random sample and therefore these findings may not generalize to other populations. Second, this study is based on self-reported information and is susceptible to recall bias and socially desirable responding. We anticipate that any response bias would likely underestimate the prevalence of risk behaviors and therefore bias our results towards the null.

In summary, we found that inability to access addiction treatment predicted injection initiation among street-involved youth. Facilitating engagement with addiction treatment by reducing wait lists and increasing the availability of low-threshold evidence-based treatments offer important opportunities to engage with vulnerable youth and potentially prevent them from transitioning to injection drug use.

## Abbreviations

ARYS: At-Risk Youth Study
IQR: Interquartile range
CI: Confidence Interval
HR: Hazard ratio
